# Left-Sided Pseudomonas Infective Endocarditis Presenting with Septic Shock and Ischemic Stroke in a Non-Injection Drug User Receiving PD-L1 Inhibitor Therapy

**DOI:** 10.7759/cureus.113961

**Published:** 2026-08-04

**Authors:** Gilgamish Maloul, Arnav K Singla, Nelish Ardeshna, Sean Swearingen

**Affiliations:** 1 Internal Medicine, Rush University Medical Center, Chicago, USA; 2 Cardiology, Rush University Medical Center, Chicago, USA

**Keywords:** central venous catheter, durvalumab, immune checkpoint inhibitor, immunotherapy, infective endocarditis, injection drug use, mitral valve vegetation, pd-l1 inhibitor, pseudomonas aeruginosa

## Abstract

Infective endocarditis from *Pseudomonas aeruginosa* is a rare but serious infection with high morbidity and mortality. We report the case of a 66-year-old female with small cell lung cancer and no history of injection drug use or structural heart disease who developed left-sided infective endocarditis from *Pseudomonas aeruginosa*. Her risk factors for this severe infection included healthcare exposure, a central venous catheter, malignancy, and recent PD-L1 inhibitor therapy with durvalumab. She was ultimately deemed not a surgical candidate and, given her poor prognosis, her family elected for discharge to home hospice with palliative therapy. This case highlights the high morbidity of *Pseudomonas *endocarditis despite combination antipseudomonal antibiotics and the shifting epidemiology of *Pseudomonas *endocarditis to left-sided infection in non-injection drug users with complex comorbidities, affecting surgical candidacy. We also discuss whether immunotherapy may represent an additional, hypothesis-generating risk factor for serious infections such as *Pseudomonas *endocarditis.

## Introduction

Infective endocarditis (IE) is a highly morbid infection of the cardiac endothelium that can have a wide range of presenting symptoms varying in clinical severity, anywhere from persistent low-grade fevers to septic shock and ischemic stroke. The most common organisms that cause IE include *Staphylococcus aureus*, viridans streptococci, and coagulase-negative staphylococci. IE caused by *Pseudomonas* *aeruginosa* is a rare but highly aggressive infection associated with significant morbidity and mortality, with overall mortality rates >30%. Historically, *Pseudomonas* IE was predominantly described among persons who inject drugs and typically involved the right-sided cardiac valves. However, more recent reports suggest an epidemiologic shift toward healthcare-associated infections and left-sided valvular involvement in older patients with multiple comorbidities [1,2]. Left-sided *Pseudomonas* endocarditis is particularly devastating given its association with embolic complications, antimicrobial resistance, and overall poor clinical outcomes.

Immunosuppression is an important risk factor for IE. Immune checkpoint inhibitors (ICIs) have transformed the management of multiple malignancies and continue to gain expanding indications. Since the FDA approval of ipilimumab in 2011 for patients with metastatic melanoma, the estimated eligibility for ICIs among patients in the U.S. with 20 general tumor types rose from 1.54% to 55.47% in 2023 [3]. There are currently four classes of ICIs named by their targets: anti-CTLA-4, anti-PD-1, anti-PD-L1, and anti-LAG-3. These immune checkpoints serve as co-inhibitory receptors to allow for self-tolerance and prevent autoimmunity. Many cancers exploit these pathways to avoid immune surveillance. ICIs block these targets, restoring T-cell-mediated antitumor immunity. Therefore, they are not typically thought of as immunosuppressive. Although infections in patients receiving ICIs are attributed to steroids or other immunosuppressive therapies used to treat immune-related adverse events, emerging literature suggests that checkpoint pathway modulation may also alter immune responses in ways that are not fully understood, which increase atypical infection risk [4].

We report a rare case of left-sided *Pseudomonas* IE in a patient with small cell lung cancer receiving durvalumab, a PD-L1 inhibitor, who presented with acute ischemic stroke and septic shock. This case highlights the evolving epidemiology of *Pseudomonas* endocarditis, the fulminant nature of left-sided disease, and the potential role of immunotherapy as a risk factor for severe infections.

## Case presentation

The patient is a 66-year-old female with a past medical history of metastatic small cell lung cancer on immunotherapy with durvalumab, prior stroke with residual hemiparesis and dysphagia requiring chronic gastrostomy tube, type 2 diabetes, and prior myocardial infarction who presented with altered mentation and reported low-grade fevers.

History obtained from the patient's family members revealed three days of physical decline with decreased level of alertness and activity. They also noted "episodes" of diaphoresis and patient discomfort after flushing of her right upper extremity peripherally inserted central catheter (PICC), which had been placed two months prior to receive ICI therapy. She had been hospitalized two months prior for a similar presentation and was treated for multifocal pneumonia as well as acute cystitis from group B streptococcus. She was on cycle 13 of durvalumab, receiving her first infusion 16 months ago, and her scheduled infusion four weeks prior had been held due to concern for infection. She had no known history of intravenous drug use, valvular pathology or prosthetics, intracardiac device, or heart failure with reduced ejection fraction.

**Figure 1:**
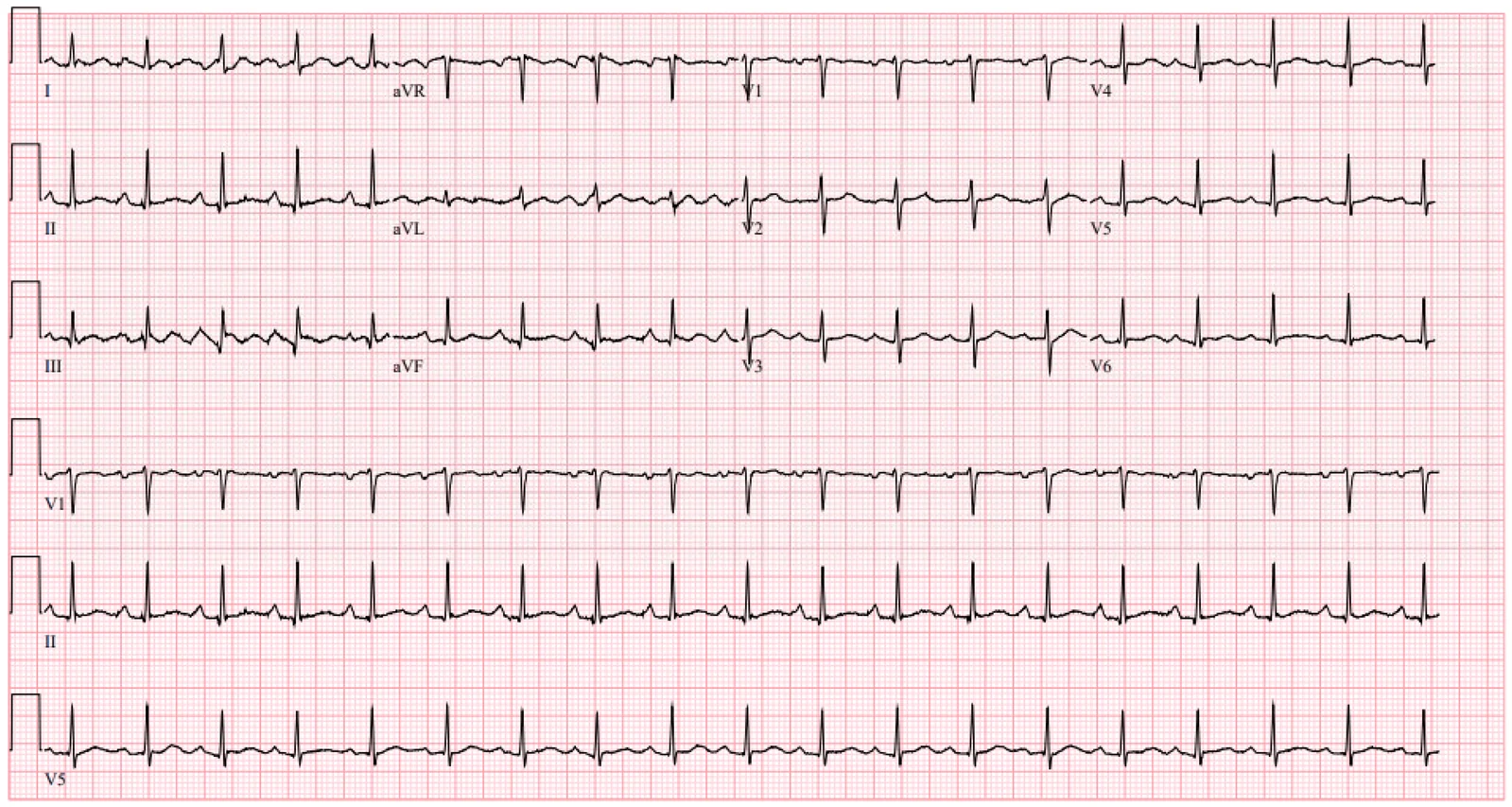
Electrocardiogram with sinus tachycardia

**Figure 2:**
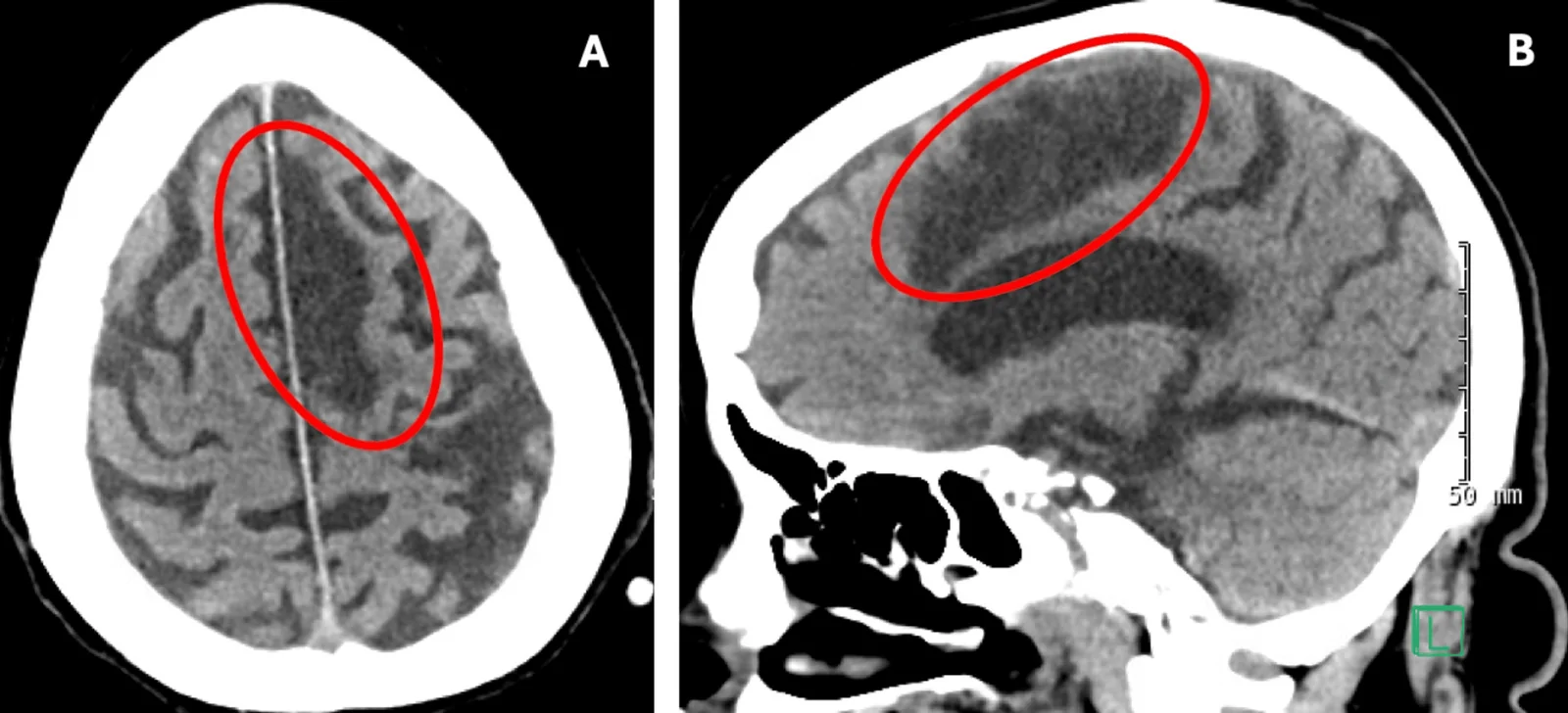
CT head without contrast revealing a large area of hypoattenuation in the left paramedial frontal lobe (red circle) in the (A) axial view and (B) sagittal view. When compared to a prior CT head, this was concerning for evolution of a prior ischemic stroke

**Figure 3:**
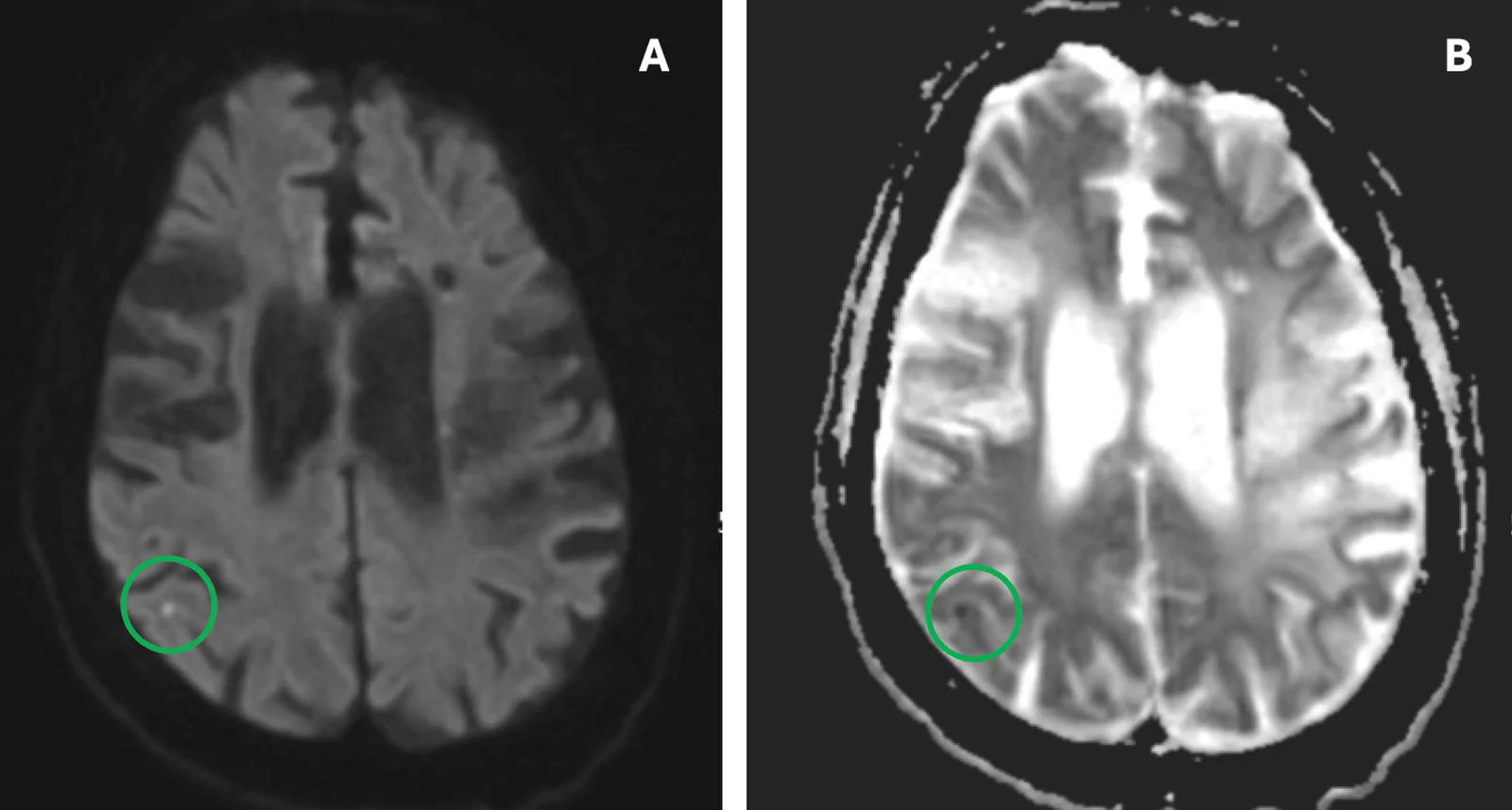
MRI brain with and without contrast demonstrating acute punctate infarct A) Diffusion-weighted imaging demonstrating a punctate area of restricted diffusion in the right parietal cortex (green circle). (B) The corresponding apparent diffusion coefficient (ADC) map shows low ADC signal in the same region (green circle), confirming a new acute ischemic stroke.

On presentation, the patient was septic with hypotension unresponsive to fluids and requiring norepinephrine, tachycardia, and a fever of 102F. On exam, she was ill-appearing, somnolent, and responsive only to painful stimuli. Pulmonary exam revealed rhonchi at the right base; cardiac exam did not reveal an obvious murmur. No Janeway lesions, Osler nodes, or Roth spots were present. Her neurologic exam revealed left-sided neglect. The EKG showed sinus tachycardia without acute ischemic changes or atrioventricular nodal delay (Figure 1). A chest X-ray showed a right lower lobe consolidation. Her initial labs revealed a leukocytosis to 20.2 k/UL (reference 4.0 - 10.0k/UL), an acute kidney injury with a creatinine of 1.35 mg/dL (reference 0.65 - 1.00 mg/dL), and a high-sensitivity troponin I level within normal limits at 10.0 ng/L (reference 0.0 - 14.0 ng/L). A stroke workup was initiated, including negative testing for syphilis and HIV. CT scan of the head without contrast showed temporal evolution of a prior infarct in the left frontal lobe (Figure 2), and an MRI brain with and without contrast revealed a new punctate infarct in the right parietal lobe (Figure 3), as well as chronic infarcts in bilateral middle cerebral artery territories, deep nuclei, and right cerebellum. Prior to discharge, a repeat CT scan of the head without contrast was obtained and showed no interval change.

**Figure 4:**
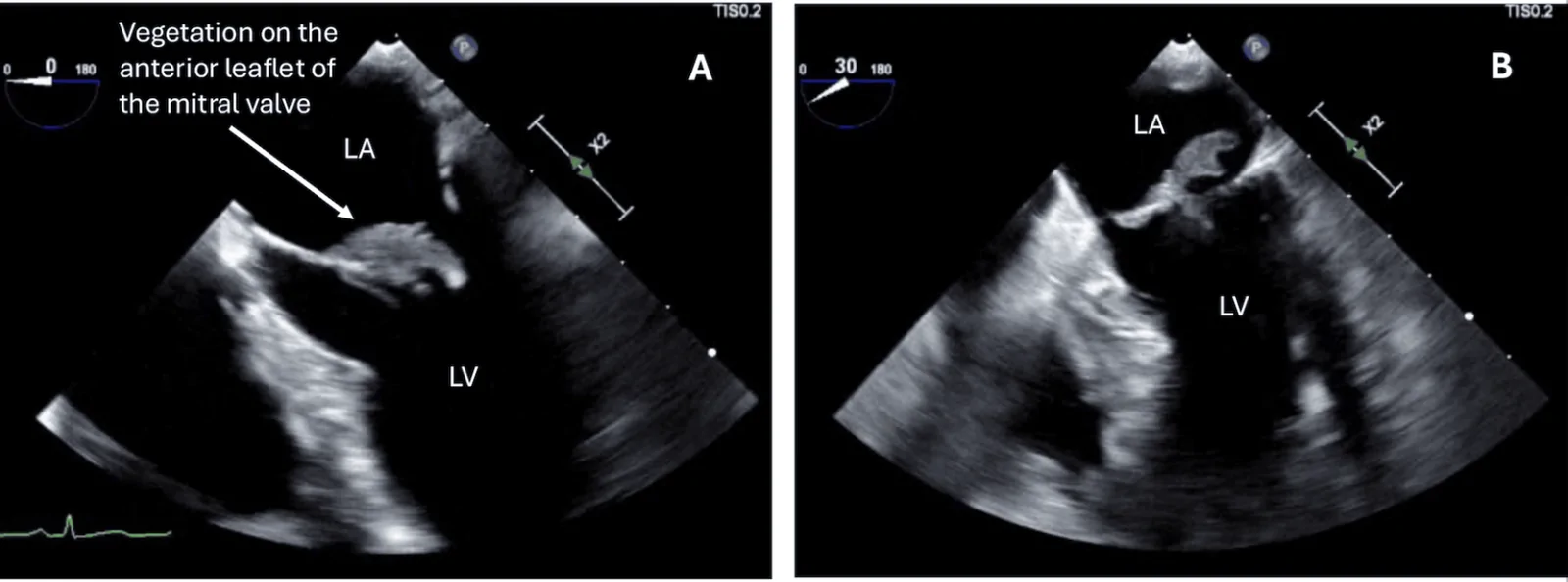
TEE in the mid-esophageal long-axis view with a large, mobile vegetation on the atrial aspect of the anterior leaflet of the mitral valve (A) during diastole and (B) systole, confirming the diagnosis of infective endocarditis TEE - transesophageal echocardiogram; LA - left atrium; LV - left ventricle

On admission, a broad infectious workup was also completed, and she was started on empiric antibiotics with vancomycin and piperacillin-tazobactam. A viral respiratory panel was negative, and sputum cultures were negative. On day two, a transthoracic echocardiogram with bubble study was obtained, revealing an ejection fraction of 70-75%, no evidence of intracardiac shunt, but it was a limited study for evaluation of the valves due to body habitus. A transesophageal echocardiogram (TEE) was planned to rule out infective endocarditis. Her blood cultures on the day of admission grew pan-susceptible *Pseudomonas aeruginosa*, and daily surveillance cultures for the subsequent two days were persistently positive. Given her high-grade bacteremia and possible involvement of the central nervous system, on day five of admission, after three sets of positive blood cultures, antibiotics were switched to cefepime and high-dose tobramycin for dual anti-pseudomonal coverage. Her hemodynamics improved, and she was weaned off pressor support, but she continued to have a fever. Her fourth set of blood cultures, drawn approximately 96 hours after her initial positive blood cultures, would ultimately show clearance. On day five, her peripherally inserted central catheter (PICC) was removed, and cultures of the catheter tip were obtained, but there was no growth. Her PICC may still have been the source of infection, and four days of antibiotics prior to removal may have resulted in a negative culture. Later that day, a TEE was performed and revealed a large, mobile vegetation measuring 2.0 cm by 1.0 cm on the atrial aspect of the anterior leaflet of the mitral valve with trivial regurgitation (Figure 4), confirming the diagnosis. She underwent prompt surgical evaluation for mitral valve replacement for definitive source control but was unfortunately deemed not a surgical candidate due to her baseline poor functional status and comorbidities including metastatic cancer, baseline hemiparesis, frailty, and dysphagia with G-tube dependence. On day six, given her poor prognosis and no option for surgical intervention, after discussions with her family, she was ultimately discharged to home hospice on palliative levofloxacin to reduce bacteremia-related symptoms.

## Discussion

This patient met criteria for a definitive diagnosis of IE based on persistent *Pseudomonas* *aeruginosa *bacteremia and echocardiographic evidence of mobile mitral valve vegetation with additional supportive features of fever and acute ischemic infarct consistent with vascular phenomenon. *Pseudomonas* *aeruginosa* is recognized as a typical organism of IE only in the setting of intracardiac prosthetic material. Notably, the classic skin manifestations of IE (Osler nodes and Janeway lesions) are often absent in *Pseudomonas* endocarditis [5], as was the case with our patient.

Prompt recognition of *Pseudomonas* IE is particularly important given its substantial mortality. In a contemporary case series, the 30-day mortality rate was 13%, increasing to 20% at six months and 60% at five years, with one-year mortality more common in patients with advanced age, significant comorbidities, left-sided valvular involvement, and complications of infection [1]. This highlights the importance of early diagnosis and timely initiation of appropriate antimicrobial therapy.

Several risk factors have been associated with *Pseudomonas* IE, including healthcare exposure, injection drug use, prior cardiac surgery or prosthetic cardiac devices, age greater than 60 years, urinary tract instrumentation, central venous catheters and other intravascular devices, and immunosuppression [6, 7]. Historically, *Pseudomonas* IE was considered a disease primarily affecting persons who inject drugs and was most associated with right-sided valvular infection. However, recent data suggest a shifting epidemiology. A contemporary cohort study found that only 29% of patients with *Pseudomonas* IE reported injection drug use [2]. Walczak et al. reported left-sided involvement in 60% of cases of *Pseudomonas* IE in their modern case series [1]. Our patient represents another example of this evolving paradigm with left-sided involvement and no history of injection drug use. Her risk factors included healthcare exposure, a central venous catheter, metastatic malignancy, and noteworthy exposure to an ICI; while associated with infectious complications, the mechanism and independent risk of ICI exposure remains debated. 

During our patient's visit, she was evaluated by infectious disease specialists, cardiologists, and cardiothoracic surgeons. Involvement of a multidisciplinary team is crucial to achieve the best possible outcomes. Current recommendations for treatment of *Pseudomonas* IE include prompt initiation of combination antimicrobial therapy consisting of an anti-pseudomonal β-lactam agent, such as cefepime, piperacillin-tazobactam, ceftazidime, or ceftolozane-tazobactam, together with either an aminoglycoside, typically high-dose tobramycin, or a fluoroquinolone for a total duration of six weeks. Importantly, medical therapy alone rarely results in the cure of left-sided *Pseudomonas* IE, and combined medical and surgical management provides the greatest likelihood of survival [8]. However, surgical intervention also carries significant risk. A large, multicenter retrospective study estimated early mortality risk following surgery for IE at 11% (298/2715), and *Pseudomonas* infection was shown to be independently associated with early surgical mortality (OR 4.33; 95% CI 1.35 - 13.93; p=0.014) [9]. The major reported risk factors, recommended treatment strategies, and outcomes of *Pseudomonas* IE are summarized in Table 1. With the inherent risks of surgery and the apparent shift in demographics of *Pseudomonas* IE to an older and more morbid population, the gap between surgical indication and actual surgery rates may grow. As it pertains to our patient, while ischemic stroke is not a barrier to surgery, stroke prior to surgery is an independent predictor of not undergoing surgery for IE (OR 0.54; 95% CI 0.32 - 0.90) [10].

**Table 1:** Summary of reported risk factors, recommended treatment, and outcomes of Pseudomonas aeruginosa infective endocarditis

	Summary	Key references
Risk factors	Healthcare exposure, injection drug use, prior cardiac surgery or prosthetic cardiac devices, age >60 years, urinary tract instrumentation, central venous catheters or other intravascular devices, and immunosuppression.	[6,7]
Recommended antimicrobial therapy	Six weeks of combination therapy with an anti-pseudomonal β-lactam (cefepime, piperacillin-tazobactam, ceftazidime, or ceftolozane-tazobactam) plus either high-dose tobramycin or a fluoroquinolone.	[8]
Role of surgery	Left-sided Pseudomonas IE is rarely cured with medical therapy alone. Combined medical and surgical management provides the highest likelihood of survival when feasible. IE surgery carries substantial perioperative mortality (around 11% overall). Pseudomonas infection is independently associated with early surgical mortality.	[8,9]
Clinical outcomes	High mortality despite modern therapy. Contemporary series reported 30-day mortality of 13%, 6-month mortality of 20%, and 5-year mortality of 60%. Worse outcomes are associated with advanced age, significant comorbidities, left-sided disease, and infectious complications.	[1]

A unique aspect of this case is the occurrence of *Pseudomonas* IE in a patient receiving immunotherapy. Our patient was receiving durvalumab infusions for small cell lung cancer. Traditionally, infectious complications in patients receiving ICIs were attributed primarily to corticosteroids or other immunosuppressive therapies administered for immune-related adverse events. However, emerging evidence suggests that ICIs themselves may exert direct immunosuppressive effects by dysregulating immune pathways involved in infection surveillance and pathogen clearance [4]. Supporting this concept, Karam et al. conducted a retrospective study of 200 patients treated with anti-PD-1 or anti-PD-L1 agents and found that infections following ICI therapy were not associated with concomitant corticosteroid or immunosuppressant use [11]. These findings raise the possibility that checkpoint inhibition may independently alter antimicrobial immunity, predisposing susceptible patients to infections from pathogens such as *Pseudomonas aeruginosa*.

## Conclusions

*Pseudomonas* *aeruginosa* is a rare cause of infective endocarditis. There is a changing epidemiology of *Pseudomonas* IE, which is increasingly encountered with left-sided involvement and in older patients without injection drug use but with healthcare-associated risk factors and complex comorbidities. *Pseudomonas* IE is a highly morbid condition that requires early identification with echocardiography, a multidisciplinary approach, and prompt treatment with combination antibiotics and evaluation for early surgical intervention. As the use of immune checkpoint inhibitors continues to expand, further investigation is needed to clarify their potential role as an independent risk factor for bacterial infections from pathogens including *Pseudomonas aeruginosa*.
